# Epitope Binning of Novel Monoclonal Anti F1 and Anti LcrV Antibodies and Their Application in a Simple, Short, HTRF Test for Clinical Plague Detection

**DOI:** 10.3390/pathogens10030285

**Published:** 2021-03-02

**Authors:** Adva Mechaly, Einat B. Vitner, Yinon Levy, David Gur, Moria Barlev-Gross, Assa Sittner, Michal Koren, Haim Levy, Emanuelle Mamroud, Morly Fisher

**Affiliations:** 1 The Department of Infectious Diseases, Israel Institute for Biological Research, Ness-Ziona 7410001, Israel; einatv@iibr.gov.il (E.B.V.); moriab@iibr.gov.il (M.B.-G.); assas@iibr.gov.il (A.S.); michalk@iibr.gov.il (M.K.); haiml@iibr.gov.il (H.L.); 2 The Department of Biochemistry and Molecular Genetics, Israel Institute for Biological Research, Ness Ziona 7410001, Israel; yinonl@iibr.gov.il (Y.L.); gurd@iibr.gov.il (D.G.); emmym@iibr.gov.il (E.M.)

**Keywords:** plague, F1, LcrV, monoclonal antibodies, HTRF, immuno-detection

## Abstract

Mouse monoclonal antibodies were raised against plague disease biomarkers: the bacterial capsular protein fraction 1 (F1) and the low-calcium response—LcrV virulence factor (Vag). A novel tandem assay, employing BioLayer Interferometry (BLI), enabled the isolation of antibodies against four different epitopes on Vag. The tandem assay was carried out with hybridoma supernatants, circumventing the need for antibody purification. The BioLayer assay was further adopted for characterization of epitope-repetitive antigens, enabling the discovery of two unique epitopes on F1. The selected antibodies were purified and applied as “oligo-clonal” reagents for the immuno-detection of both biomarkers. The developed Homogenous Time Resolved Fluorescence (HTRF) tests were short (10 min) and simple (no washing steps), allowing for detection of 10 ng/mL F1 and 2.5 ng/mL Vag. The tests were successfully applied for detection of disease biomarkers produced by various *Y. pestis* strains during growth in blood culture vials.

## 1. Introduction

The growing interest in rapid diagnostic tools for medical use (e.g., clinical and point-of-care diagnostics) has encouraged the development of fast, simple-to-operate immunoassays. One such assay is the Homogeneous Time Resolved Fluorescent (HTRF) assay, which was recently applied in our laboratory for the detection of anthrax, botulinum and plague [1,2]. This homogeneous immunoassay is a lanthanide based Förster Resonance Energy Transfer (FRET) assay that does not require any wash steps. The assay mixture is comprised of specific antibodies labeled with either a donor or an acceptor fluorophore. The efficiency of the donor-acceptor energy transfer is inversely proportional to the sixth power of the distance between both molecules [3]. Accordingly, both fluorophores have to be in close spatial proximity (<10 nm) to achieve efficient FRET [4]. This only occurs when both antibodies interact with the target molecule, resulting in a positive signal that is proportional to the antigen concentration. 

An efficient HTRF assay requires the implementation of high affinity antibodies that interact with different epitopes on the designated antigen. Hence, once a large panel of positive hybridoma clones is identified, one has to determine the affinity and epitope coverage (epitope binning) of the entire panel in order to correctly pair the assay’s antibodies. This process is both costly and laborious. While many groups established various high-throughput binning schemes [5,6,7,8,9,10,11], most require purified antibodies. In other cases, where hybridoma supernatants were characterized directly, anti-mouse antibodies were utilized to capture and screen the antibodies [8,10], thereby introducing an isotype bias. We developed a strategy, based on Bio-Layer Interferometry (BLI), that enabled the selection of high affinity antibodies (based on *koff* ranking) against different epitopes directly from hybridoma supernatants, bypassing the need for antibody purification during the characterization process. 

As a proof of concept, we chose to generate and screen antibodies against plague soluble biomarkers: the pathogen’s capsule protein fraction 1 (F1) and the LcrV (Vag) virulence protein. Throughout history, plague led to the estimated death of millions of people [12] and it continues to be endemic in various areas in the world, mainly Africa, China, the U.S. South-West and the Russian Federation (CDC Website). Plague’s pathogen, *Yersinia pestis*, is categorized as a Tier 1 select agent [13], highlighting the need for specific assays that will enable rapid and accurate detection at the earliest possible stage of infection. Detection of F1, as a soluble protein in clinical specimens, has been reported as a reliable indication of plague infection [14,15,16,17]. Recently, we reported that Vag, which is a part of the type III secretion system of *Y. pestis*, could also be used as a soluble disease biomarker for the early detection of plague [18]. This additional biomarker is especially important for detection of atypical virulent *Y. pestis* strains that do not produce the F1 antigen [19,20,21,22].

By implementing the developed screening methodology, we were able to perform epitope binning directly from hybridoma supernatants, identifying four separate epitopes on Vag. We further adapted our methodology to enable the epitope binning of repetitive antigens (such as F1), resulting in the elucidation of two distinct epitopes on F1. The resulting antibodies were successfully applied to develop HTRF homogeneous immunoassays for the detection of both Vag and F1.

## 2. Results

### 2.1. Antibody Generation and Screening

Monoclonal antibodies against the recombinant *Yersinia pestis* antigens F1 and Vag (rF1, rVag) were generated and screened by either the conventional ELISA-screening method or by a flow cytometry-based screening method [23]. Positive antibody clones were further characterized, as described in the following sections, in order to identify high affinity antibodies targeting diverse epitopes on both antigens

### 2.2. Epitope Binning of Anti-Vag Antibodies

In order to develop a binning strategy, we applied the BLI technology. We started by selecting one highly active, ELISA positive, anti-Vag clone. This antibody (denominated 129) was purified, biotinylated (Bio-129) and used to scan all other anti-Vag antibodies in a classic pair-wise mapping scheme. In this format, simultaneous binding of the tested antibodies induces a wavelength shift in the interference pattern, which indicates that the two tested antibodies bind non-overlapping epitopes. In contrast, if the two antibodies bind the same or partially-overlapping epitopes, no or a low wavelength shift is induced. Antibody 129, used as a control in these experiments, did not react with Bio-129 bound Vag, indicating that the soluble Vag is mostly monomeric. During this analysis, all anti-Vag antibodies (except Bio-129) were implemented as culture hybridoma supernatants. 

**Figure 1:**
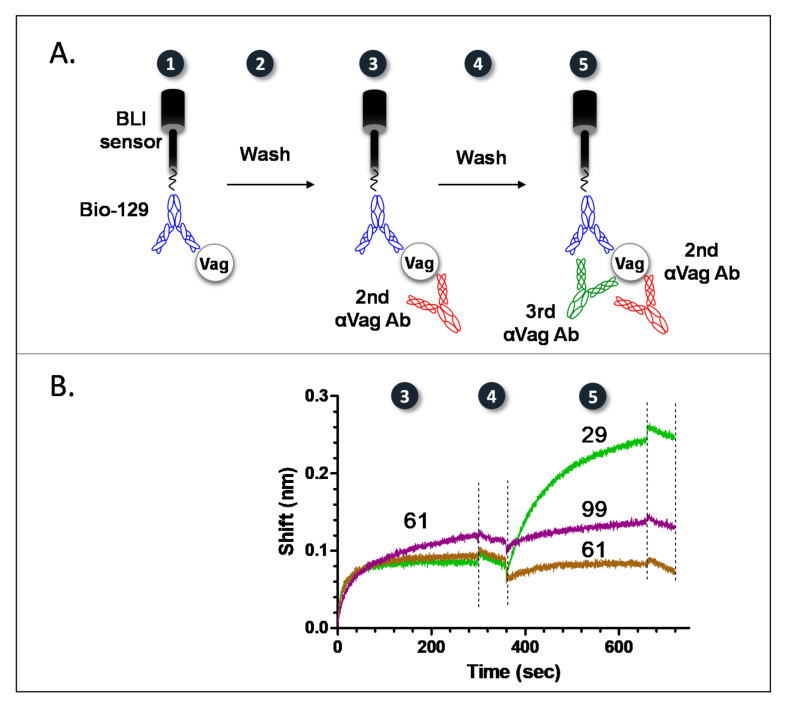
A tandem methodology for epitope binning of anti-Vag antibodies. (**A**) A schematic representation of the devised tandem methodology. After the capture of a purified biotinylated anti Vag antibody (Bio-129) by the streptavidin BLI sensor, the assay consisted of antigen (Vag) capture (step 1), followed by a wash step (step 2). The complex was then reacted (step 3) with a 2nd anti Vag antibody (until saturation), followed by an additional wash step (step 4), and probed with a 3rd anti Vag antibody (step 5). (**B**) Implementation of the devised tandem methodology for epitope mapping of anti-Vag clones 61 (brown line), 99 (purple line) and 29 (green line). Numbers (in black circles) mark the various binding and washing steps, as described in the (**A**) section. All antibody clones, except Bio-129, were implemented as hybridoma supernatants (diluted 1:1 in Octet buffer). Wash steps are indicated by dashed lines.

At the end of this classic scan, antibodies that bound the same epitope as 129 were put aside (in a single “bin”) and the “bin” of the remaining antibodies was assigned utilizing a novel, tandem, scheme, schematically depicted in Figure 1A. 

As indicated, Vag was reacted with sensor-bound Bio-129 (blue antibody) (step 1). The complex was then washed (step 2) and reacted with a 2nd antibody (red antibody) (step 3). After an additional wash step (step 4), the resulting complex was finally reacted with a 3rd antibody (pink antibody) (step 5). A shift in the interference pattern (at step 5) would indicate that the 3rd antibody binds a different epitope than both the 1st and 2nd antibodies. Since the 3rd antibody was previously determined (using the pairwise scheme described) as interacting with a different epitope than 129, no shift would indicate that the 3ed antibody reacts with the same epitope as the 2nd antibody. This “three-way” scheme hinges on the fact that the 2nd antibody must reach saturation, i.e., all of its binding sites must be blocked. Figure 1B depicts an actual binding experiment (only steps 3 to 5 are presented), where antibodies 29 (green) and 99 (magenta) are characterized concerning their binding epitope, compared to antibody 61 (brown). In this scheme, antibody 61 is the secondary antibody and antibodies 29 or 99 are the tertiary antibodies (both secondary and tertiary antibodies are implemented as culture hybridoma fluids). As can be seen, antibody 99 binds the same epitope as antibody 61 (no shift in interference pattern is observed in step 5, magenta line), while antibody 29 interacts with a different epitope than both antibody 61 and antibody 129 (a visible shift in interference pattern is seen in step 5, green line). As a control, antibody 61 was implemented as both the 2nd and the 3rd antibodies (brown line). The fact that no interference shift was observed indicates that all the binding sites of the 2nd antibody were indeed saturated. The same sensor was then regenerated and used with the same secondary antibody to map all additional antibodies in a similar manner, thus creating a second “bin”. This “three-way” binning methodology was iteratively used, applying a different secondary antibody for each iterative step, to map all the antibodies. 

This methodology enabled us to assign the 33 anti-Vag antibodies to four different bins. The four bins included 2, 4, 9 and 15 antibody clones, respectively, with 129, our “handle” antibody, being part of the second largest bin. Three of the anti-Vag antibody clones (out of a total of 33 clones isolated) presented a very weak interaction and thus were not mapped into any bin. 

### 2.3. Picking the Best Anti Vag Antibody from Each Bin 

In order to select the best representative antibody from each bin, without actually determining antibody affinity, we took advantage of the fact that the BLI technology enables on-line monitoring of *kon* and *koff* for each antibody. As opposed to *kon*, which is time and concentration dependent (1/M·s), *koff* is only time depended (1/s) and can therefore be determined even when the antibody concentration is unknown. Hence, comparing the *koff*s of the different antibodies provides information regarding the antibody’s KD. This methodology was implemented by others to select antibodies from diverse pools [24,25]. The researchers observed a strong correlation between antibody affinity and antibody off-rates (R^2^ = 0.82), while no observable correlation existed between affinity and on-rates (R^2^ ≤ 0.03) [24]. Using this criterion, we chose antibodies displaying the slowest *koff* rates from each bin, thus replacing antibody 129 with antibody 59. The selected hybridomas from each bin (denominated: 38, 59, 107 and 122) were used to generate ascetic fluids in mice, and the resulting antibodies were purified. 

### 2.4. Characterization of Anti Vag Antibodies

**Figure 2:**
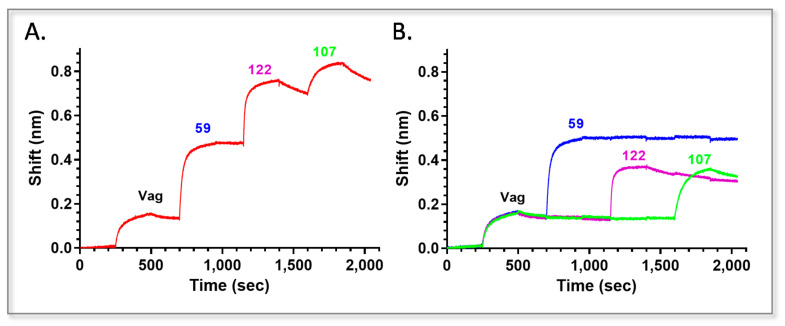
Consecutive binding of anti-Vag antibodies. The ability of the different anti Vag antibodies to bind (**A**) consecutively or (**B**) separately to Vag was tested using the Octet Rad biolayer interferometry system. Biotinylated antibody 38 was captured by the streptavidin sensor (this step is not depicted) and loaded with Vag. The 38-Vag complex was then interacted with the indicated antibodies separately or concomitantly.

In order to implement all the selected antibodies in Vag detection, we verified that the purified antibodies could bind the antigen simultaneously, using the Octet Red biolayer interferometry system. To this end, purified anti-Vag antibody 38 was biotinylated (Bio-38) and captured by a streptavidin sensor. After a short interaction with Vag, antibodies 59, 122 and 107 (the chosen antibodies from each bin based on their *koff*s) were reacted consecutively with the 38-Vag complex (Figure 2A). As a control, each antibody was reacted separately with the same complex (Figure 2B). As can be seen, all the antibodies were able to bind Vag simultaneously. The binding of the final antibody (107) is somewhat hindered, displaying a lower interference shift (65%) compared to that achieved by the same antibody that was reacted separately with the antigen, indicating that some stearic hindrance may occur (applying the nine possible sequential combinations using Bio-38 as the handle indicated that when 107 was implemented as the second antibody, the binding of the following two antibodies was not hindered).

We next determined the affinity constants of the selected anti Vag antibodies. Each antibody was biotinylated, immobilized on a streptavidin sensor and monitored for its Vag binding profile at different antigen concentrations. The sensorgrams were fitted with a 1:1 binding model, and the association (*kon*) and the dissociation (*koff*) constants were determined (Table 1). 

The affinity of the four antibodies ranged between 3.1 nM for 107 and a 10-fold higher affinity of 300 pM for 59. The correlation between *koff* and affinity was indeed demonstrated for antibody 59, which displayed the lowest *koff,* and accordingly, the highest affinity. However, for antibody 107, a low *kon* rate resulted in a somewhat lower affinity, despite the improved *koff* demonstrated by the antibody.

### 2.5. Epitope Binning of Anti-F1 Antibodies

**Figure 3:**
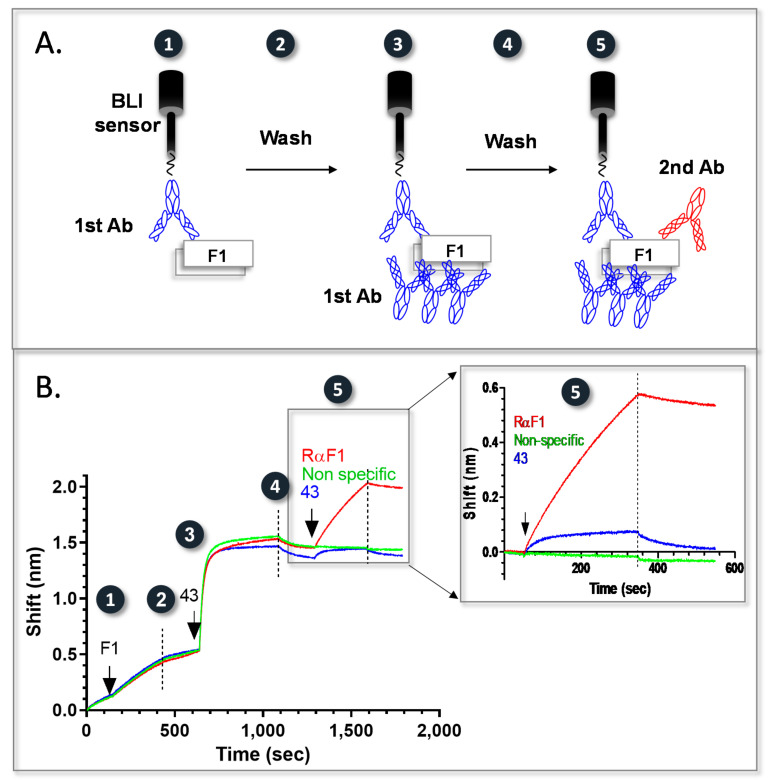
Pairwise mapping of antigens displaying repetitive epitopes. (**A**) A schematic representation of a pairwise mapping approach for F1. A streptavidin BLI sensor coated with a purified biotinylated anti F1 primary antibody is used to capture F1 (step 1). Following a wash step (step 2), the complex is saturated with the same unlabeled primary antibody (step 3) and following an additional wash step (step 4), probed with a 2nd anti F1 antibody (step 5). (**B**) Assay calibration. The numbers depicted on the calibration assay correspond to the numbers on the schematic representation. For assay calibration, three BLI streptavidin sensors were coated with anti F1 Bio-43. After F1 capture (step 1), the resulting complexes were saturated with unlabeled 43 (step 3) and the different sensors were reacted (step 5) with either a non-specific antibody (green line), antibody 43 (blue line) or a rabbitαF1 polyclonal antibody (red line). Wash steps are indicated with dashed lines. The calibration assay was carried out with IgG purified antibodies.

The F1 capsular antigen of *Y. pestis* is comprised of homo-polymers that include many F1 monomeric units [26,27]. Since the binning process depends on the singularity of the monoclonal antibody–antigen interaction, the repetitive nature of this antigen posed a challenge, compared to the anti-Vag antibodies binning. Thus, as schematically depicted in Figure 3A, we implemented a different approach. Following F1 capture by a purified, biotinylated “handle” antibody (Ab 43), all of the antibodies’ additional binding sites were blocked (by immersing the complex with the same, non-biotinylated antibody until the sensogram reached saturation) and only then probed for a possible interaction with a secondary antibody. This strategy was examined in a preliminary test, as portrayed in Figure 3B. Three streptavidin sensors were reacted with the biotinylated, ELISA positive, “handle” antibody (Bio-43). Following F1 binding (step 1), all additional 43 binding sites were blocked (step 3), creating complex 43-F1-43. The individual sensors were than incubated (step 5) with the same concentration of the indicated, IgG purified, antibodies: 43 (blue line), polyclonal rabbit αF1 (red line) or a mouse monoclonal nonspecific antibody (green line). As expected, there was no interaction of the blocked complex with the non-specific antibody, while a significant interaction was observed with the polyclonal rabbitαF1. An unexpected small interaction was observed when 43 was used as a secondary antibody (since all its binding sites were blocked and the curve reached saturation). This small apparent interaction did not disappear even when a prolonged incubation with a higher concentration of 43 was implemented. 

**Figure 4:**
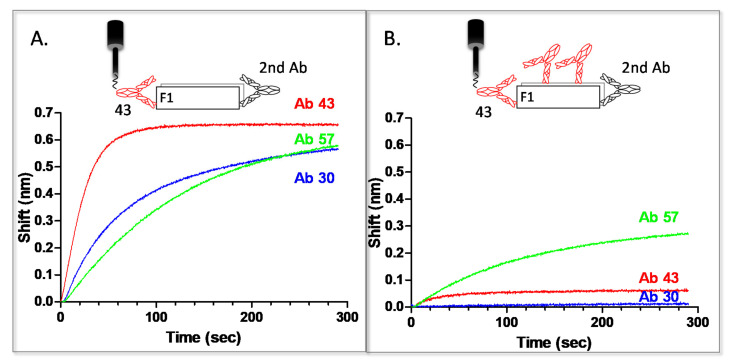
Pairwise mapping of anti F1 antibodies. Hybridoma supernatants containing anti F1 antibodies: 43 (red), 57 (green) or 30 (blue) were reacted with (**A**) F1 antigen captured by 43 or (**B**) F1 antigen captured and saturated by 43 (biotinylated or unlabeled, respectively).

Due to this slight interaction, the devised methodology for anti-F1 hybridomas characterization included a comparison of the binding amplitude for each probed antibody with or without blocking of the Bio-43-F1 complex (with antibody 43) as follows: streptavidin sensors were interacted with Bio-43 and used to capture F1. This complex was initially reacted with a second anti-F1 hybridoma supernatant with no blocking step, to ensure that the antibody is functional and to determine the maximal amplitude of the observed reaction. The same sensor was then regenerated and the procedure was repeated, this time with a blocking step prior to the interaction with the secondary antibody. Results for antibodies 30 and 57 are presented in Figure 4. Only the binding step (step 5 in Figure 3) of the secondary antibody is presented.

As can be seen in Figure 4A, all three antibodies reacted with the F1 that was captured by 43 (when no blocking step was applied). However, after blocking all of antibody’s 43 binding sites, only 57 interacts with the antigen, indicating that this antibody binds a different epitope than 43 (Figure 4B). The interaction is somewhat hindered (the observed amplitude after blocking is half of that detected when 43 sites are not blocked), probably due to some stearic interference caused by the abundance of blocked 43 binding sites on F1. Another possibility is that the antibodies bind overlapping epitopes. It is interesting to point out that, while 30 did not exhibit any interaction with F1 after blocking all of its binding sites (which overlap antibody 43’s binding sites), 43 continued to display the unexplained slight interaction previously observed. This might indicate that those two antibodies bind to a similar, but not identical binding site. Of the examined anti F1 antibodies, only 57 reacted with a different epitope than 43, resulting in the isolation of two antibodies against two different (though possibly overlapping) epitopes on F1. The additional antibody (57) was propagated and purified. Due the oligomeric nature of the F1 antigen, the affinity of the anti F1 antibodies was not determined. 

### 2.6. HTRF Test for Antigen Detection 

For Vag and F1 detection, we chose a FRET based HTRF test that was previously developed in our laboratory [28]. The test is short (10 to 30 min), simple and requires no washing steps (the test is extensively described in the introduction section). Results are presented as ΔF signals (representing energy transfer) that are calculated, as described in the materials and methods section.

**Figure 5:**
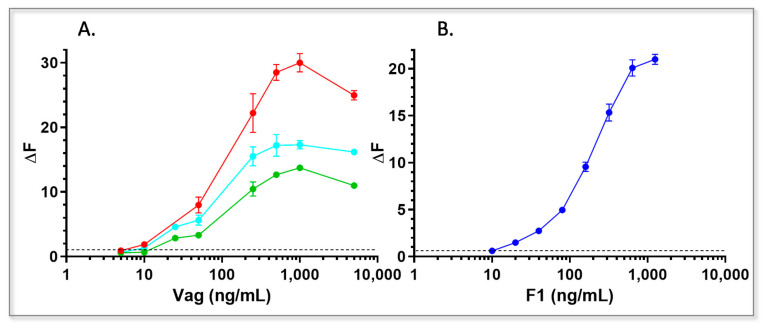
Dose response curves of HTRF immunoassays for detection of spiked soluble disease bio-markers in PBS. PBS was spiked with different concentrations of (**A**) rVag or (**B**) rF1. Antigen samples were examined with the following tests: For Vag detection: 59-Loisto615, 38-Alexa647 and 122-Alexa647 (red), 59-Loisto615, 38-Alexa647 (cyan) or 59-Loisto615, 122-Alexa647 (green) and for F1 detection: 43-Loisto615, 57-Alexa647 (blue). Points are averages of at least three independent sets of measurements. ΔF ratios were calculated as described in materials and methods and the limit of detection for each test was determined for ΔF ratios ≥ 0.5 (Black dashed line).

For assay calibration, purified antibodies were conjugated to either donor or acceptor fluorophores and applied in the test at different concentrations. For Vag detection, we initially tested the performance of all antibody pairs, followed by the analysis of different antibody combinations (“pseudo” polyclonal, i.e., oligo-clonal). The best oligo-clonal combination, demonstrating the highest sensitivity and dynamic range, constituted of antibody 59 as the donor antibody, combined with antibodies 122 and 38 as acceptor antibodies (Figure 5A, red) at a donor-acceptor ratio of 1:6. This test demonstrated a synergistic effect when compared to the paired assays (Figure 5A, cyan and green). It is important to point out that all the tests (in all of the combinations tested) were adversely affected when any attempt to combine more than one donor was made (regardless of monoclonal antibody identity or affinity). This might be due to quenching effects resulting from the close proximity of the donor antibodies.

The monoclonal-based assay was able to detect as little as 2.5 ng/mL of rVag (assay detection limit was calculated as described in the Material and Methods and was established as ΔF ≥ 0.5, black dashed lines in Figure 5) in clean samples (PBS) with a dynamic range of more than two orders of magnitude. For F1 detection, we analyzed the performance of antibodies 43 and 57 as both donor and acceptor conjugates. The best antibody combination consisted of 43 as a donor and 57 as an acceptor at a donor-acceptor ratio of 1:10. This test enabled detection of 10 ng/mL rF1 (ΔF ≥ 0.5) in clean samples (PBS) with a dynamic range of two orders of magnitude. 

As described, the developed HTRF tests are homogenous, i.e., specific antibody conjugates are mixed with the unknown sample (antigen) and analyzed directly with no washing steps. As a result, one can actually measure the signal repeatedly, at different times, without impeding assay progression. Analysis of the developed teats after 10, 20 or 30 min indicated that there is almost no difference in the assays’ detection limit, regardless of incubation time (Figure S1). A time dependent improvement in the dynamic range was observed, especially for the anti F1 test (see Figure S1: red curve vs. blue curve), indicating a possible slower *kon* for the anti F1 antibodies in comparison to the anti-Vag antibodies. The results of both assays indicate that they can be analyzed as soon as 10 min after sample addition with no significant change in LOD. 

### 2.7. Detection of Disease Biomarkers from Inoculated Blood Cultures

We next wanted to implement the developed tests for antigen detection in clinical samples, such as blood cultures. During the assay calibration phase (described above), PBS was used as the control sample for the HTRF signal (ΔF) calculation. We have previously demonstrated that, for complex matrices, such as blood cultures, one cannot use PBS (non-spiked control buffer) as the control due to significant differences in fluorescent emission between PBS and blood [2]. In these cases, the unknown blood sample can be used as its own control, by analyzing the same sample with two separate assays: A specific test and an internal control test (in which only the specific donor is applied, coupled with a non-specific acceptor of the same antibody isotype). To this end, internal control tests were calibrated for both Vag and F1 assays and applied for antigen detection from spiked blood. The observed LOD in blood cultures was similar to that achieved in PBS (example of experimental results for F1 detection are presented in Supplemental Material Figure S2). 

### 2.8. Universality of Antigen Detection 

We next wanted to verify that the developed assays could be applied for native antigen detection, i.e., detection of disease biomarkers produced by *Y. pestis* during growth (in the blood culture vial). To this end, several *Y. pestis* strains were inoculated into blood culture vials containing naïve human blood and grown at 37 °C for 48 h. Bacterial counts were determined and the presence of native F1 or Vag in the supernatants of the blood cultures was analyzed. Results are presented in Table 2. The final bacterial concentration for each probed culture (at the end of the 48-h incubation) is presented using a color code, with color intensity correlating with higher bacterial concentration (light green to dark green). A similar color code was applied to ΔF values that are presented for each antigen (yellow to dark orange or light to dark blue for ΔF value for F1 detection or Vag detection, respectively) and correlate with antigen concentration that was calculated based on calibration curves performed in the same medium (Figure S2).

As can be seen, the developed tests detected the native antigens in a range of 2–500 ng/mL and 0.003–2 µg/mL for Vag and F1, respectively, from *Y. pestis* strains grown to 1 × 10^6^–1 × 10^8^ cfu/mL. All undiluted samples were found to be positive, with no false negative results. Strains lacking the pCD1 plasmid (carrying the gene coding for Vag expression) were not recognized by the Vag HTRF test, acting as internal controls, indicating the validity and specificity of the developed test. The results also indicate that the cutoff for biomarkers detection was around 1–2 × 10^6^ cfu/mL (For the Oreintalis biovar).

### 2.9. Specificity of Antigen Detection 

All human pathogenic *Yersinia* species express the V-antigen, however, intraspecific diversity exist. To determine the specificity of the test, several *Y. entrocolitica* (O:3 IP134, O:8 ATCC27729 and O:9 IP383) and *Y. pseudotuberculosis* (484337) strains were inoculated into blood culture vials and tested for the presence of F1 and Vag, in two separate experiments. As expected, no signal was detected by the F1 assay as these strains do not express F1. However, at high bacterial concentrations, the Vag assay detected the *Y. pseudotuberculosis* strain (at 3 × 10^8^ cfu/mL) as well as the *Y. enetrocolitica* strains (at 1 × 10^9^ cfu/mL). None of the tested strains were recognized at lower concentrations (5 × 10^7^ cfu/mL and lower), demonstrating between two and three orders of magnitude difference in antigen detection between Vag from *Y. pestis* and that of other *Yersinia* strains. Since neither *Y. enetrocolitica* nor *Y. pseudotuberculosis* cause pneumonia and are both gastrointestinal pathogens (presenting an entirely different clinical picture compared to *Y. pestis*), this will likely not lead to a misdiagnosis (false positive). Notably, the possibility of bacteremia resulting from *Y. entropathogens* infection still exists in rare cases. This wide range recognition might present an advantage, since the test is more likely to detect a verity of *Y. pestis* strains [29]. We further characterized assay specificity by testing additional pneumonia causing Tier-1 select agents such as *F. tularensis*, *B. anthracis* or the common hospital-acquired bacteria *P. aeruginosa* as well as additional gram-negative bacteria of the family Enterobacteriaceae such as *S. typhimerium* and *E. coli*. No signals were detected, even at high bacterial concentrations (1 × 10^8^–1 × 10^9^ cfu/mL), indicating the high specificity of the developed tests. 

## 3. Discussion

The aim of this study was to develop a diverse collection of high affinity monoclonal antibodies, targeting different epitopes on both Vag and F1 and the implementation of the developed antibodies in short, simple, HTRF tests for quick, specific detection of plague in clinical samples. 

To this end, we generated 33 hybridomas against Vag and 5 hybridomas against F1. We sought a methodology that will enable the organization of the antibody panels into distinct epitopic families/bins (epitope binning) without the need for antibody purification. For this purpose, we devised a novel tandem methodology (employing BLI technology) that led to the allocation of different anti-Vag antibodies into four distinct bins. Within each bin, we used the same information, acquired during the above-mentioned binning experiments, to select the lowest *koff* value-demonstrating antibody for further purification and characterization. These antibodies, representing the four different epitopes discovered on Vag, demonstrated simultaneous binding to the antigen, enabling the theoretical creation of an oligo-clonal mixture for antigen detection. For F1 detection, we adopted a different approach (using the same technology), tailored to facilitate epitope binning of antibodies produced against repetitive-epitope antigens. This methodology enabled the discovery of antibodies against two distinct epitopes on F1.

In the past, several groups developed monoclonal antibodies against both Vag and F1. These antibodies were applied for antigen detection and protection [14,30,31,32,33,34,35,36,37,38,39]. While the isolation of monoclonal antibodies against two epitopes on F1 antigen was previously demonstrated [40], the work presented herein is the first to report the isolation of monoclonal antibodies against four distinct epitopes on Vag. Thus, this enlightened the simplicity of the developed methodology for quick and simple scanning of antibody panels. 

We next attempted the formation of a characterized, reproducible, oligo-clonal antibody formulation with the intention of generating a possible alternative for polyclonal antibody preparations, which are finite and prone to non-specific interactions. In this respect, one has to consider whether an oligo-clonal formulation, consisting of a definite number of antibodies, can indeed be a good substitute for a polyclonal antibody, namely, how many epitopes are actually present in a polyclonal antibody reagent. In an attempt to answer this question, we previously characterized the epitope repertoire of a polyclonal antibody reagent against the ricin toxoid, by screening against three, commercially available phage-display peptide libraries [41]. The results indicated that the polyclonal reagent reacted with four unique epitopes on the toxin. In a later work, we constructed a phage display library from monkeys immunized with the same toxin. Again, monoclonal antibodies were isolated against a total of four distinct epitopes on both ricin sub-units [42]. In another work, where monoclonal antibodies were generated against abrin toxin, we discovered a total of five unique epitopes on abrin’s two sub-units [43]. In both cases, the phage-display libraries were iteratively screened, using antibodies representing previously isolated epitopes, until no unique epitopes emerged. In the literature, others attempted to answer the same question, discovering between two to nine epitopes on different peptides, cytokines, cell-surface receptors and enzymes [7,8,10]. In another, very comprehensive work, the authors generated complex node plots of deduced bin assignments for several antigens, screening hundreds of antibodies [6]. The authors reported up to twenty-five different functional bins on one of the antigens characterized, however, many represented over-lapping epitopes (resulting in a total of nine non-overlapping bins). In conclusion, it is logical to assume that an oligo-clonal reagent comprising four to seven epitopes will ensure wide-range detection. 

The anti-Vag and anti-F1 antibodies were implemented in a homogeneous, one-step, time-resolved-fluorescence based test (HTRF test), for short (10 min), specific detection of plague infection. The anti-Vag test consisted of antibodies against three of the four discerned epitopes, while both novel antibodies (against two discrete epitopes on the F1 antigen) were incorporated in the anti-F1 test. The developed assays recognized all *Y. pestis* strains tested (Table 2), exhibiting high specificity (no cross-reaction was observed with other pneumonia-causing bacteria such as *B. anthracis*, *F. tularensis* or *P. aeruginosa* that were grown in blood culture vials that obtained final bacterial concentrations of >10^8^ cfu/mL). 

The tests enabled detection of 2.5 ng/mL Vag or 10 ng/mL F1 from both PBS and blood culture samples (within 10 min). These detection limits are relevant for plague detection from human clinical samples, as it was previously established that measured levels of F1 in patients with bubonic plague are in the range of 4 ng/mL and 50 µg/mL [31]. In another study involving 194 patients, it was determined that the geometric mean concentrations of F1 in bubo fluids, serum and urine were around 70 ng/mL, 40 ng/mL and 15 ng/mL, respectively [14], all of which are expected to be positively identified by our developed test. As for Vag detection, we have previously established a mouse model for both bubonic and pneumonic plague [18]. In these models, Vag concentrations in the serum ranged between a few to dozens ng/mL, 48 h post infection, indicating that most of the infected animals would have been identified in the new HTRF test. The LODs demonstrated by our tests are similar to some previously published tests [14,30,34,44] while being inferior to others [32,40,45]. However, at least for Vag detection, some of the published tests that demonstrated higher sensitivity [32], were multi-step assays that were carried out within one to two and half hours. The major advantage of the newly developed tests presented in this study is that the application of both, simultaneously, provides built-in confirmation and reduces the likelihood of both false negative and false positive results. Since the existence of F1-negative *Y. pestis* virulent strains is documented [19,21,22], it is of importance to enable *Y. pestis* detection via both F1 and Vag. We have previously shown that our tests can be lyophilized with no loss of activity, enabling transportation and handling at room temperature [46]. This might facilitate direct application of the tests in plague endemic areas.

In summary, in this work, we developed novel monoclonal antibodies against four distinct epitopes on Vag and 2 epitopes on F1. The antibodies were initially characterized as hybridoma fluids using novel schemes implementing the BLI technology. The antibodies were incorporated in simple, one step HTRF tests, enabling sensitive, rapid detection of both F1 and Vag, plague disease biomarkers from complex clinical samples.

## 4. Materials and Methods

### 4.1. Ethical Statement

This study was performed in accordance with the recommendations for the Care and Use of Laboratory Animals (National Institutes of Health [NIH]). Animal experiments were performed in accordance with Israeli law and were approved by the Institutional Ethics Committee for animal experiments at the Israel Institute for Biological Research (Permit number: IACUC-IIBR M-08-2015, M-58-2017). 

### 4.2. Antigens

Generation of recombinant F1 (rF1) was described previously [47,48]. Recombinant V antigen (Vag) was produced and purified, as described in References [49,50].

### 4.3. Strains

The bacterial strains used in this study are listed in Table 3.

**Table 3:** Bacterial strains used in this study.

Strains	Relevant Characteristics	Reference
***Yersinia*** **strains**		
***Yersinia pestis***		
Kimberley53 (Kim53)	Virulent strain. Biovar Orientalis	[51]
Kimberley53 ∆pCD1∆pPCP1	Spontaneously pPCP1 and pCD1-cured Kimberley53	[52]
Bombay∆pCD1	Biovar Orientalis	[53]
Alexander	Virulent strain. Biovar Orientalis	[54]
EV76	*pgm*− (Girard’s strain). Biovar Orientalis	[55]
IV 75 195	Virulent strain.	This study
KIMD27Δ*pgm*	Biovar Mediaevelis	[56]
***Yersinia pseudotuberculosis***	484337	[57]
***Yersinia enterocolitica***		
IP134	O:3	[58]
ATCC27729	O:8	[59]
IP383	O:9	
**Other strains**		
*E. coli*	ATCC25922	
*P. aeruginosa*	ATCC27853	
*S. typhimurium*		
*B. anthracis*	Vollum ATCC 14578 (Tox+ Cap+)	Bacillus Genetic Stock center
*F. tularensis holarctica*	ATCC29684	

### 4.4. Immunization

Female BALB/c mice (Charles-River, initial weight of 18–20 g) were immunized with a mixture of rVag and rF1 (50 µg each). Three sequential subcutaneous vaccinations (a priming dose followed by two boosts, 100 µL/dose) were carried out every 21 days. One group (*n* = 4) was primed with complete Freund’s adjuvant (Sigma, Rehovot, Israel) and boosted with incomplete Freund’s adjuvant (Sigma, Israel). The second group (*n* = 4) was primed and boosted with antigens mixed with Alum (0.36% final concentration). A 4th intravenous boost of both antigens without adjuvants was administered 3 days before sacrifice (15 weeks from the 3rd injection). Blood samples were taken prior to each dose and IgG titers were determined by ELISA. Three days after the last boost, the chosen mouse (exhibiting the highest titers for both antigens) was sacrificed and hybridomas were generated (there was no significant difference between the final titers observed for the two adjuvants).

### 4.5. Hybridoma Generation

Splenocytes from immunized mouse were fused with mouse myeloma NS0 cells (ECACC no. 85110503), as described in Reference [60]. The obtained hybridoma clones were screened by either ELISA against plate adsorbed antigens (rF1 or rVag) or by a newly developed flow cytometry screening methodology [23]. Positive clones demonstrating the highest immune-reactivity, were further cloned by limiting dilution, resulting in a total of 33 clones and 5 clones that were isolated for Vag and F1, respectively. For generation of hybridomas in mice, cultured hybridoma cells (5 × 10^6^–1 × 10^7^) were injected i.p. into (C57BL/6 X BALB/c) F1 adult mice that had been treated 7–30 days previously with an i.p. injection of 2,6,10,14-tetramethylpentadecane (prystane). Ascitic fluid was harvested usually 10 to 20 days after hybridoma inoculation.

### 4.6. Polyclonal Antibodies Generation

Generation of hyperimmune rabbit polyclonal antibodies against rF1 and rVag was described previously [61]. The immunoglobulin G (IgG) fractions were purified from the hyperimmune sera using HiTrap Protein G (GE Healthcare, Uppsala, Sweden) according to the manufacturer’s instructions.

### 4.7. Antibody Labeling

Biotinylation of IgG purified antibody fractions was carried out using sulfo-NHS-SS-biotin [sulfosuccinimidyl-2-(biotinamido) ethyl-1,3-dithiopropionate; Pierce 21331] according to the manufacturer’s instructions. The number of biotin moieties per antibody has been calculated using the HABA ([2-(4-hydroxyazobenzene] benzoic acid) method (Pierce 28050) and was shown to be four (in average). IgG purified antibody fractions were also labeled with Loisto615 using Loisto615 Europium Chelate ITC-activated (Abacus Diagnostica Oy, Turku, Finland) and with AlexaFluor 647 (A647) using Molecular Probes^®^ Alexa Fluor^®^ antibody labeling kit (ThermoFisher Scientific, Waltham, MA, USA), according to the manufacturer’s instructions.

### 4.8. Biolayer Interferometry

Antibody binding studies were carried out using the Octet Red system (ForteBio, Version 8.1, Menlo Park, CA, USA, 2015). All steps were performed at 30 °C with shaking at 1500 rpm in a 96-well plate containing 200 µL solution in each well. PBS buffer, pH 7.4, containing 10 mg/mL BSA and 0.1% (v/v) Tween 20 was used throughout this study for antibody and analyte dilution and for washing the sensors. Streptavidin-coated biosensors were loaded with a mouse anti Vag/F1 biotinylated antibody (5 µg/mL) for 300 s followed by a 60 s wash. The sensors were then reacted for 300 s with LcrV/F1 (5 µg/mL), washed again for 60 s and submerged in wells containing the concentrated hybridoma medium of the different antibodies for another 300 s followed by another 60 s wash. As a positive control, a poly clonal anti Vag/F1 antibody was used [61]. Medium from a non-related mouse mono-clonal hybridoma was used as a negative control in the assay.

### 4.9. Preparation of Blood Cultures

*Yersinia* strains were grown on BHIA (Brain heart infusion agar, BD Difco, Sparks, MD, USA, #241830) plates at 28 °C for 48 h. *E. coli*, *P. auraginosa* and *S. typhimurium* were grown at 37 °C for 24 h. Colonies were suspended in sterile phosphate buffered saline (PBS, Biological industries, Beth haemek, Israel, #02-023-1A) and added at a defined concentration into BACTEC plus aerobic/F culture vials (BD, MD, USA, #442192) supplemented with 10 mL of naïve human blood containing Citrate-phosphate-dextrose solution with adenine (CPDA) as an anticoagulant. The inoculated blood culture vials were then shaken at 150 rpm at 37 °C in a New Brunswick Scientific C76 water bath for different durations. Final colony forming units (CFU) counts for each vial were determined by plating 0.1 mL of serial 10-fold dilutions on BHIA plates and incubating for 48 h at 28 °C. *F. tularensis* subsp. *holarctica* vaccine strain LVS and *B. anthracis* Vollum strain were grown, as described in Reference [62]. The resulting blood cultures were processed, as described in the next section.

### 4.10. Blood Culture Processing

For detection of soluble disease bio-markers, blood cultures were centrifuged (14,000× *g*, 5 min) and the supernatant was applied directly in the assay. Supernatants form virulent strains growth were filtered (0.22 µm) and sterility was verified by plating.

### 4.11. HTRF Assays

Assays were performed in white (non-maxisorb) microplates (Nunc, Roskild, Denmark) in duplicates. Specific antibodies conjugated to Loisto615 or AlexaFluor-647 (A647) were implemented in the assays as the donor and acceptor, respectively. Donor and acceptor antibodies, diluted in 20 µL assay buffer (1% BSA (Israel Biological Industries, Kibbutz Beit-Haemek, Israel), 5% trehalose (Sigma T9531)), were incubated with 40 µL analyte (diluted in PBS or blood) for 10, 20 or 30 min. Results were read in the Infinite F200 reader (Tecan, Männedorf, Switzerland) twice, in the following settings: 1. Excitation with 340 nm (±35 nm) and emission at 612 nm (±10 nm) and 2. Excitation with 340 nm (±35 nm) and emission of 665 nm (±8 nm). The lag time was 100 μs and integration time was 400 μs. Excitation of the Loisto615 donor results in an energy emission at 612 nm that causes the excitation of the acceptor molecule only when the acceptor is in close proximity (d < 10 nm) to the donor. The emission of the Alexa647 acceptor at 665 nm is proportional to antigen concentration.

### 4.12. Calculation of HTRF Signals

The HTRF signals (ΔF) were calculated as normalized fluorescence transfer signals [63] using the following formula:

(1) $$\Delta F=\;\frac{\frac{F\left(665\;nm\right)Sample}{F\left(612\;nm\right)Sample}-\frac{F\left(665\;nm\right)control}{F\left(612\;nm\right)control}}{\frac{F\left(665\;nm\right)control}{F\left(612\;nm\right)control}}$$

In the formula, the average fluorescence intensity of the sample acceptor (Ab-Alexa647) at 665 nm, i.e., "F(665 nm)Sample", was divided by the fluorescence intensity of the sample donor (Ab-Loisto615) at 612 nm i.e., "F(612 nm)Sample". The same ratio, calculated for the control, was subtracted from the sample ratio and the result was then divided by the control ratio. Two types of controls were used in this study: reference to blank (PBS) or reference to an internal control test (An additional test, where only the specific donor was applied, coupled with a non-specific acceptor). To evaluate the limit of detection (LOD), the average of background ΔF readings was calculated for each assay as the mean response of at least six “noise” (PBS) samples. The LOD was defined as five standard deviations (SDs) above the average background with a coefficient of variation (CV) < 15%. These values yielded a signal (noise + 5 SD)-to-noise (average noise), which was considered to be the LOD threshold. This value was lower than 0.5 for all tests. For simplicity in data presentation and comparison, a value of ΔF ≥ 0.5 was set as the positive threshold for all assays in accordance with ICH guidelines for validation of the analytical procedures [64]. This calculation enabled the normalization of multiple experiments and the determination of a universal threshold for positive samples. 

### 4.13. Safety Considerations

*B. anthracis*, *Y. pestis*, and *F. tularensis* have been classified as tier one select agents. In the United States, possession, use, storage, or transfer of tier 1 organisms requires approval of the Centers for Disease Control and Prevention (CDC) Select Agent Program. Handling of these select agents is subject to select agent regulations and should be carried out in a biosafety level 3 (BSL3) laboratory, according to the international guidelines for the use and handling of pathogenic microorganisms. *B. anthracis* and some strains of *Y. pestis* were handled according to the above-mentioned regulations. Notably, in this study, we used LVS as a model for *F. tularensis* and other attenuated or BL2 strains, which are exempt from select agent regulations in the United States (https://www.selectagents.gov/SelectAgentsandToxinsExclusions.html). As for the BSL2 strains, the work was performed in a BSL2 laboratory. 

## Figures and Tables

**Table 1:** Affinity constants of purified anti-Vag antibodies.

Ab	*Kon* (1/M·s)	*Koff* (1/s)	K_D_ (nM)
59	1.4 ± 0.3 × 10^6^	3.6 ± 0.4 × 10^−4^	0.3 ± 0.2
122	1.2 ± 0.03 × 10^6^	1.1 ± 0.4 × 10^−3^	0.9 ± 0.2
38	1.6 ± 0.09 × 10^6^	4.4 ± 0.8 × 10^−3^	2.8 ± 0.5
107	1.9 ± 0.5 × 10^5^	6.0 ± 0.3 × 10^−4^	3.1 ± 1.0

**Table 2:** Universal recognition of F1 and Vag produced by different *Y. pestis* strains.

*Y*. *pestis* Strains	Biovar	Bacterial Concentration (cfu/mL)	F1	Vag	Estimated Antigen Concentration	
			ΔF F1	ΔF Vag	F1 (ng/mL)	Vag (ng/mL)
EV 76	Orientalis	2.5 × 10^7^	28.1	19.2	876	485
Kimberly53 *		4.7 × 10^6^	90	10	1000	248
		2.0 × 10^7^	274.4	9.8	1504	242
Kim53ΔpCD1ΔpPCP1**		2.0 × 10^7^	2262	-	15,637	-
BombayΔpCD1 **		1.3 × 10^8^	233.9	-	1216	-
Alexander *		2.3 × 10^6^	14.4	4.9	388	115
IV 75 195 *		1.2 × 10^6^	2.7	1.1	73	15
KIMD27Δ*pgm*	Mediaevalis	1.4 × 10^8^	4.1	0.6	21	2

* Virulent *Y. pestis* strains. ** This strain does not produce Vag due to loss of the pCD1 plasmid. Blood culture samples were tested undiluted or 10- and 100-fold diluted. ΔF values were calculated from the linear range. Antigen concentrations were calculated using calibration curves obtained with the recombinant antigens. Colors: Light to dark green, yellow to dark orange, Light to dark blue: bacterial concentrations and ΔF values for F1 or Vag detection, respectively, from low to high.

## Supplementary Materials

The following are available online at https://www.mdpi.com/2076-0817/10/3/285/s1: Figure S1: Time dependent detection of HTRF tests, Figure S2: Dose response curves of recombinant F1 in different media.
