# Supplementary material for: Epitope Binning of Novel Monoclonal Anti F1 and Anti LcrV Antibodies and Their Application in a Simple, Short, HTRF Test for Clinical Plague Detection
Source: Pathogens. 2021 Mar 2;10(3):285. doi: 10.3390/pathogens10030285 (PMC8001648; doi:10.3390/pathogens10030285)
Supplement: Supplementary file 1 [file pathogens-10-00285-s001.pdf]

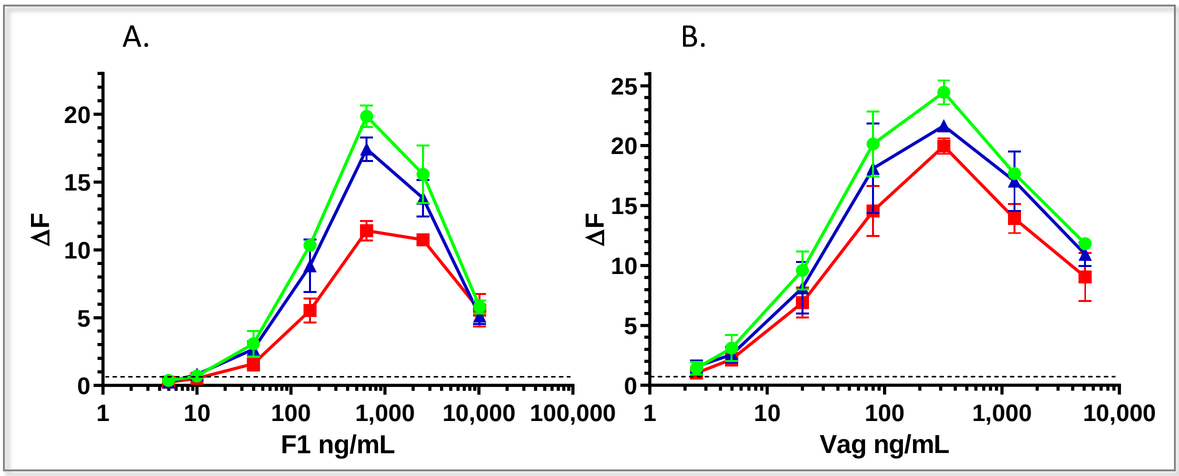

Figure S1. Time dependent detection of HTRF tests. Donor and acceptor antibodies for (**A**) F1 detection, (**B**) Vag detection were incubated with different concentration of rF1 (5 ng/mL–10 µg/mL) or rVag (2.5 ng/mL–5 µg/mL) respectively. Tests were read after 10 (red), 20 (blue) and 30 (green) minutes.

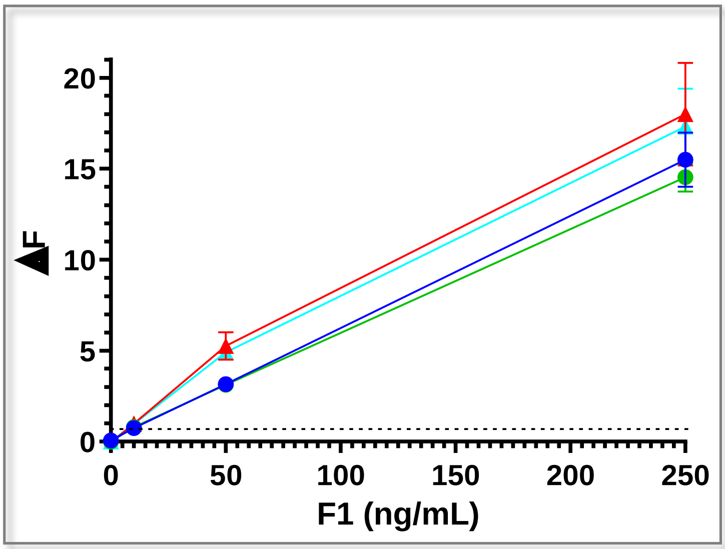

Figure S2. Dose response curves of recombinant F1 in different media. rF1 was spiked in PBS (circles) or in blood culture bottles containing blood from healthy human donors (triangles). The samples were examined simultaneously with both the specific and the internal control tests and ΔF signals were calculated either with the un-spiked sample as control (green and cyan) or with the internal control signal of each sample as control (blue and red).
